# A core set of patient-reported outcomes for population-based cancer survivorship research: a consensus study

**DOI:** 10.1007/s11764-020-00924-5

**Published:** 2020-08-31

**Authors:** Imogen Ramsey, Nadia Corsini, Amanda D. Hutchinson, Julie Marker, Marion Eckert

**Affiliations:** 1 Rosemary Bryant AO Research Centre, UniSA Clinical & Health Sciences, University of South Australia, City East Campus, GPO Box 2471, Adelaide, South Australia 5001 Australia; 2 UniSA Justice & Society, University of South Australia, Adelaide, South Australia Australia; 3 Cancer Voices South Australia, Adelaide, Australia

**Keywords:** Cancer survivorship, Quality of life, Patient-reported outcomes, Core outcome set, Delphi study, Consensus

## Abstract

**Purpose:**

Core outcome sets aim to improve the consistency and quality of research by providing agreed-upon recommendations regarding what outcomes should be measured as a minimum for a population and setting. This study aimed to identify a core set of patient-reported outcomes (PROs) representing the most important issues impacting on cancer survivors’ long-term health, functioning, and quality of life, to inform population-based research on cancer survivorship.

**Methods:**

In phase I, a list of 46 outcomes was generated through focus groups (*n* = 5) with cancer survivors (*n* = 40) and a review of instruments for assessing quality of life in cancer survivorship. In phase II, 69 national experts in cancer survivorship practice, research, policy, and lived experience participated in a two-round Delphi survey to refine and prioritise the listed outcomes into a core outcome set. A consensus meeting was held with a sub-sample of participants to discuss and finalise the included outcomes.

**Results:**

Twelve outcome domains were agreed upon for inclusion in the core outcome set: depression, anxiety, pain, fatigue, cognitive problems, fear of cancer recurrence or progression, functioning in everyday activities and roles, financial toxicity, coping with cancer, overall bother from side effects, overall quality of life, and overall health status.

**Conclusions:**

We established a core set of PROs to standardise assessment of cancer survivorship concerns at a population level.

**Implications for Cancer Survivors:**

Adoption of the core outcome set will ensure that survivorship outcomes considered important by cancer survivors are assessed as a minimum in future studies. Furthermore, its routine use will optimise the comparability, quality, and usefulness of the data cancer survivors provide in population-based research.

## Background

Rising cancer incidence, advancements in detection and treatment, and improved survival rates have contributed to an unprecedented number of people living with a diagnosis of cancer [1, 2], a group known as cancer survivors [3]. An increasing number of survivors are managing cancer (and its sequelae) as a chronic condition [4] as adverse long-term and late effects related to the disease and treatment can have debilitating and lifelong consequences [5]. More than 50% of adult cancer survivors experience persistent physical and functional issues that may limit ability to work and increase healthcare utilisation and costs [3, 5]. Growing interest in quantifying the impacts of illness and treatment on health, functioning, and quality of life from the patient perspective using patient-reported outcomes (PROs) has led to a proliferation in the number of patient-reported outcome measures (PROMs)—self-report questionnaires about a patient’s health—being used in clinical practice and research [6].

The potential uses of PRO can be understood using Lipscomb’s framework for cancer outcomes research, which proposes three arenas for PRO application: micro, meso, and macro [7]. At the micro or individual level, PRO data can be used to support and enhance patient-centred care, patient–clinician interactions, and clinical decision-making [6]. At the meso or service level, PROs are used to examine the variables that influence health outcomes [7]. PRO data collected at a macro level can facilitate surveillance of the impacts and burden of cancer on population health [3, 7]. There is increasing recognition that optimising population-based cancer registries to collect longitudinal, macro-level PRO data would improve understanding of trends in survivorship outcomes and their long-term trajectory [8, 9].

A challenge in PRO research is the lack of standardisation and comparability of scores from different measures [10]. Core outcome sets provide one way of addressing these problems. A core outcome set is a recommended, standardised, and minimum set of outcomes to be measured and reported in research on a specific health condition, and which should be consensually agreed to by relevant stakeholders [11, 12]. By standardising the outcomes that are examined across studies, use of core outcome sets can help to minimise potential bias in outcome selection and reporting, and enable data from different studies to be compared and synthesised [10]. Various cancer-related core outcome sets have been developed, but there is currently no core outcome set to inform assessment of long-term cancer survivorship concerns at a population level. Most existing cancer-related core outcome sets are tumour-specific and were developed to facilitate standardised assessment of acute treatment-related symptoms and toxicities in clinical trials and clinical research.

This study aimed to obtain consensus on a core outcome set, recommending what PROs should be collected as a minimum for population-based surveillance of cancer survivorship in Australia, and how these outcomes should be measured. The need for a set of outcomes applicable to all cancers is underscored by research identifying issues common across cancers including psychosocial outcomes, fatigue, functional impairment, fear of cancer recurrence, and limitations in healthcare and insurance access [13, 14].

There have been two previous efforts to standardise assessment of health outcomes across general cancer populations, which differ from this core outcome set in scope and end use. The National Cancer Institute (NCI) identified a core set of symptoms experienced by patients with cancer undergoing treatment [15], to inform standardised assessment of PROs in adult cancer treatment trials. In the Netherlands, Geerse and colleagues [16] defined a core set of categories from the International Classification for Disability, Functioning and Health (ICF) representing health-related problems experienced by adult cancer survivors more than a year from diagnosis, for an unspecified end use. Our study is the first to identify a core set of PROs relevant in long-term cancer survivorship for the purpose of routine assessment at a population level, and was developed in Australia.

Major methodological differences also separate the three studies. The initial outcome list for the NCI core set was derived from a systematic review and analysis of datasets [15], while outcomes in the study by Geerse and colleagues were derived from the ICF [16]. To obtain consensus on the NCI core set, a meeting with stakeholders was held [15]. The characteristics and expertise of the stakeholders were not reported. Geerse and colleagues conducted a Delphi study with stakeholder panels for breast, lung, and colorectal cancers to reach consensus on the most important ICF categories for survivors of these cancers in the Netherlands, then undertook a procedure to link the identified categories to existing PROMs [16]. Our approach uniquely involved consumers from the outset and identified an initial list of outcomes from focus group discussions with cancer survivors. We additionally examined cancer survivorship PROMs to ensure coverage of all outcomes assessed by validated measures. To ensure fit with the literature in terms of how PROMs are described and conceptualised, all domains in our study were classified using a PROMs taxonomy [17]. Finally, to ensure rigour, we determined consensus on the core outcomes by using comprehensive and recommended Delphi methods that were planned and published a priori [18].

## Methods

As described in the published study protocol [18], the core outcome set was developed using robust and comprehensive methods based on recommendations for core outcome set development [11] and previous studies [19, 20]. This included a Delphi process, a well-recognised method for obtaining expert consensus on a defined problem [21] that is used frequently in health research and in core outcome set development. The study design was informed by the Core Outcome Set-Standards for Design (COS-STAD) [22], which provide methodological guidance to support evidence-based core outcome set development. The study is reported in line with the Core Outcome Set-Standards for Reporting (COS-STAR) statement [23], which comprises an 18-item checklist covering the minimum reporting requirements for the sections of a paper describing the development of a core outcome set [23]. The study protocol was approved by the University of South Australia Human Research Ethics Committee (Application Number: 200370).

### Phase I: Generation of potential outcomes list

In phase I, a list of outcomes relevant to cancer survivorship was generated via focus group discussions with cancer survivors and a rapid evidence check of validated PROMs for cancer survivorship.

Between December 2017 and February 2018, 5 focus groups were held with adult cancer survivors in Adelaide and Sydney to explore their views on the impacts of cancer on well-being and everyday life. They were attended by 40 cancer survivors in total, aged between 25 and 74 years and reporting 17 different cancer types. A list of outcomes was derived from the transcribed focus group discussions. The outcomes were organised conceptually into domains informed by a taxonomy for the classification of cancer PROMs [17]. All focus group participants were sent the list and invited to provide feedback, which 6 did.

To identify any additional outcomes assessed in validated cancer survivorship PROMs, a rapid, targeted search was conducted for the highest level of evidence on measures developed to assess quality of life in adult cancer survivors. A list of multidimensional measures of quality of life developed specifically for cancer survivors was identified from the included sources. For each measure, the included domains and constructs were extracted.

To consolidate the list of outcome domains to be progressed into phase II, the domains identified in the focus groups and review of PROMs were combined and examined for clarity and duplication by the research team and consumer representatives.

### Phase II: Consensus process

#### Delphi method

To refine the list of domains generated in phase I into a core outcome set, a consensus process incorporating a three-step modified Delphi method was undertaken. The Delphi method involves eliciting the views of selected experts on a topic via anonymous sequential questionnaires. The process traditionally begins with open-ended questions that are subsequently refined through a series of rounds, interspersed with controlled feedback based on the group views, until consensus is reached [11, 21, 24]. This study utilised a modified Delphi method, limited to two questionnaire rounds and a final consensus meeting. The modifications constituted (1) beginning the process with purposely selected questions to provide a solid grounding in existing evidence and (2) including a face-to-face meeting for panel members to clarify and finalise the final core outcomes through discussion. Both modifications are consistent with recommended methodology for evidence-based core outcome set development [25]. The rationale for this approach is explained in more detail in the study protocol [18].

#### Sample and recruitment

Adult cancer survivors, oncology health professionals, and potential end users of the core outcome set with expertise in cancer survivorship research, practice, or policy were purposively sampled. Cancer survivors who partook in the focus groups were invited. Remaining stakeholders were identified from the membership directories and networks of Australian cancer survivorship organisations, committees, and research groups. All prospective participants received an email invitation with a link to register interest in participating. Online registration was open for 4 weeks, and two reminder emails were sent.

#### Round 1

In round 1, registered stakeholders received an email detailing the study purpose, a participant information sheet, and a link to the online survey. The participant information sheet explained the study background and objectives, intended end use of the core outcome set, key definitions, and ethical considerations in lay terms. The survey included questions about participants’ demographics and relevant survivorship experience or expertise, and the list of outcome domains formatted into questionnaire items with lay definitions of each term. Participants were asked to rate the importance of each item for monitoring the long-term impacts of cancer on quality of life at a population level, using the 9-point Grading of Recommendations Assessment, Development and Evaluation (GRADE) scale for the quality of evidence of outcomes [20, 26]. Scores of 7–9 indicate critical importance, scores of 4–6 indicate importance, and scores of 1–3 indicate limited importance. Space was provided for participants to comment on or explain their ratings, and to add and rate new outcomes. Two reminders were sent at weekly intervals.

#### Statistical analysis

Descriptive statistics were used to summarise the results for each round, including the median score for each item and percentage of ratings between 1 and 3, 4 and 6, and 7 and 9.

#### Feedback and round 2

Score allocations from round 1 (for each panel and overall) were presented to participants along with the link for the round 2 survey. After receiving feedback, participants were invited to re-score the outcomes from round 1 and any additional outcomes that were suggested.

#### Consensus definition

Items with a median score ≥ 7 and an interquartile range no larger than two units that received rankings of 7–9 by ≥ 70% of participants and 1–3 by ≤ 15% of participants after round 2 were eligible for inclusion in a provisional core outcome set and progressed to the consensus meeting. There are no universally agreed consensus criteria in Delphi studies; these thresholds follow published recommendations and previous core outcome set development studies in cancer [25, 27]. To ensure that outcomes considered important to individual stakeholder groups were not rejected without opportunity for reflection, items for which consensus was marginal were also discussed. Consensus was deemed marginal if an item with a median score ≥ 7 had ≥ 65% majority agreement for one panel.

#### Consensus meeting

In the round 2 survey, participants were asked to indicate their interest in attending a consensus meeting to finalise the core outcome domains. A consensus meeting was convened with a sample of 16 stakeholders representing different professions and survivorship expertise to review the results and discuss the outcomes that achieved consensus or received a marginal score. Due to the wide geographic reach of the sample, participants could attend in person or online. Following the meeting, participants were asked to vote online on a provisional core outcome set that reflected the discussion being progressed to phase III.

### Phase III: Instrument selection

This paper reports on the results from phases I and II, which established consensus on a core outcome set. Further work will be undertaken in phase III to select recommended instruments for assessment of the core outcome set [18]. Survivorship PROMs will be reviewed and evaluated applying the OMERACT Filter 2.0 [28], an instrument appraisal tool that summarises key psychometric properties. Additionally, instruments will be assessed for suitability with respect to the PRO objectives, conceptual clarity, involvement of consumers in the design, and feasibility for routine population-level assessment. The recommended instruments will be determined in consultation with potential end users of the core outcome set (e.g., registries and government agencies) and the Quality of Life Office of Cancer Australia, which provides expert advice on the design, use, analysis, and interpretation of PROs [18].

## Results

### Phase I: Identifying outcomes

The focus group discussions generated a list of 205 outcomes. The targeted search identified two comprehensive reviews of PROMs for assessing quality of life in cancer survivors, a systematic review of PROMs of the impact of cancer on survivors’ everyday lives, and a systematic review of monitoring systems that collect PROMs from cancer survivors. From these sources, 12 multidimensional PROMs developed to assess quality of life in cancer survivors were identified. The domains extracted from the PROMs were used in combination with the PROMs Cancer-Core taxonomy [17] to assist with organising the outcomes conceptually into domains.

After consolidating the list of outcome domains derived from the focus groups and review of PROMs, 46 domains remained. These were formatted into questionnaire items to be progressed into phase II.

### Phase II: Prioritising outcomes

#### Participants

A total of 195 national experts in cancer survivorship practice, research, policy, and lived experience (60 cancer survivors, 135 healthcare and research professionals) were invited to register their interest in participating in the study. Of these, 80 registered (34 cancer survivors, 46 healthcare and research professionals) and received the study information sheet and link to the online questionnaire. Sixty-nine participants (34 cancer survivors, 35 healthcare and research professionals) completed round 1 (response rate: 86%), and 54 completed round 2 (response rate: 78%). Table 1 summarises the characteristics and expertise of the participants who completed both rounds.

**Table 1:** Characteristics of Delphi participants who completed both rounds

Variable	Category	*N* (%)
Sex (*N* = 54)	Male	15 (28)
	Female	39 (72)
Age, years (*N* = 54)	18–29	0 (0)
	30–39	7 (13)
	40–49	20 (37)
	50–59	13 (24)
	> 60	14 (26)
Cancer survivor (*N* = 25)	Breast	11 (44)
	Colorectal	4 (16)
	Stomach/gastric	2 (8)
	Chronic lymphocytic leukemia	1 (4)
	Acute promyelocytic leukaemia	1 (4)
	Melanoma	2 (8)
	Neuroendocrine	1 (4)
	Peritoneal	1 (4)
	Prostate	1 (4)
	Testicular	1 (4)
	Lung	1 (4)
	Brain	2 (8)
Years since diagnosis*	< 2	0 (0)
	2–5	7 (28)
	6–10	8 (32)
	11–15	4 (16)
	> 15	6 (24)
Currently on treatment	Yes	8 (32)
	No	16 (64)
	Unsure	1 (4)
Date of last treatment	< 3 months	3 (12)
	3–12 months	2 (8)
	1–2 years	2 (8)
	2–5 years	3 (12)
	5–10 years	5 (20)
	> 10 years	6 (24)
	Treatment ongoing/not applicable	4 (16)
Health professional (*N* = 29)	Role	
	Physician	11 (38)
	Registered nurse	7 (24)
	Clinical nurse specialist	1 (3)
	Psychologist	3 (10)
	Pharmacist	1 (3)
	Speech pathologist	2 (7)
	Primary practice area	
	Haematology	3 (10)
	Medical oncology	11 (38)
	Radiation oncology	4 (14)
	Surgical oncology	1 (3)
	Radiology	1 (3)
	Symptom management/palliative care	5 (17)
	Adolescent and young adult cancer	2 (7)
	Psycho-oncology	4 (14)
	Speech pathology	1 (3)
	Disability support	1 (3)
	Rehabilitation/survivorship care	4 (14)
	General practice	1 (3)
	Other	5 (17)
	Cancers of focus	
	All cancers	13 (45)
	Breast	9 (31)
	Central nervous system	3 (10)
	Gastrointestinal	4 (14)
	Genitourinary	3 (10)
	Gynaecological	1 (3)
	Head and neck	2 (7)
	Haematological	3 (10)
	Lung	5 (17)
	Melanoma	0 (0)
	Neuroendocrine and thyroid	1 (3)
	Sarcoma	1 (3)
Cancer research experience (*N* = 54)	Clinical trials	14 (26)
	Registry-based clinical research	10 (19)
	Observational studies	15 (28)
	Epidemiology	5 (9)
	Biostatistics	1 (2)
	Psychometrics	4 (7)
	Laboratory-based research	1 (2)
	Behavioural research	14 (26)
	Health services research and evaluation	17 (31)
	Health economics	3 (6)
	Policy research	2 (4)
	Evidence synthesis	11 (20)
	Qualitative research	13 (24)

The oncology experts who participated included senior leaders of organisations and groups that provide support services to cancer survivors, facilitate cancer survivorship research and education, and inform cancer advocacy and policy. These included Cancer Australia, the Clinical Oncology Society of Australia (COSA) Survivorship Group, the Australian Cancer Survivorship Centre, Cancer Council Australia, the Psycho-Oncology Co-operative Research Group, the Quality of Life Office, the Multinational Association for Supportive Care in Cancer Survivorship Group, the Primary Care Collaborative Cancer Clinical Trials Group, Youth Cancer Services, the Peter MacCallum Cancer Centre, the Victorian Comprehensive Cancer Centre, the Queensland Collaborative for Cancer Survivorship, South Australian Cancer Services Survivorship Committee, the Western Australian Cancer Network Survivorship Collaborative, Cancer Voices South Australia, and Cancer Voices New South Wales.

In addition to demonstrating leadership in cancer survivorship, participants also had relevant clinical and research expertise. The sample included healthcare professionals from survivorship research groups (e.g., the Survivorship and Living Well After Cancer Research Program at Peter MacCallum Cancer Centre), stakeholders in national cancer survivorship policy and research priority-setting activities (e.g., the 2018 COSA PRO Monitoring Think Tank), and invited speakers from conferences (e.g., the COSA Cancer Survivorship 2019 Conference). Identifying experts through these channels meant that most health professionals (90%) were also experienced in conducting PRO research with cancer survivors. The most commonly reported area of clinical focus was medical oncology (38%) followed by symptom management/palliative care (17%), radiation oncology (14%), rehabilitation/survivorship care (14%), psycho-oncology (14%), and haematology (10%; see Table 1 for all areas). Nearly half of the health professionals reported that their practice covered all cancer types (45%). Commonly identified cancers of focus were breast (31%), lung (17%), gastrointestinal (14%), central nervous system, genitourinary, and haematological (all 10%; see Table 1 for all cancer types).

The cancer survivors reported a range of common, rare, solid, and liquid tumours, with breast cancer being the most prevalent type (44%) followed by colorectal (16%), gastric, brain, and melanoma (all 8%; see Table 1). All survivors were over 2 years from diagnosis, and most were not on active treatment (64%) (Table 1).

#### Round 1

After round 1, 38 PRO domains met the consensus criteria. Seven additional outcomes were suggested and incorporated into the questionnaire for round 2.

#### Round 2

After round 2, seven outcome domains met the consensus criteria and eight domains received marginal scores that warranted discussion. Table 2 summarises the round 2 results, showing how the domains were finally scored by each panel and the entire cohort, with the results expressed as proportions.

**Table 2:** Score allocations from round 2 by panel and total (%) critical ratings (i.e., between 7 and 9)

Outcomes	Score allocations by panel^1^						Total critical ratings (*n* = 54) (%)	Median (IQR)	Consensus
	Cancer survivors (*n* = 25)			Health and research professionals (*n* = 29)					
	Score								
	Not important (%)	Important (%)	Critical (%)	Not important (%)	Important (%)	Critical (%)			
Depression	0	0	100	0	0	100	100	9 (1)	In
Anxiety	0	12	88	0	0	100	94	8 (2)	In
Overall symptom burden	0	3	88	0	12	97	93	8 (2)	In
Pain	0	8	92	0	7	93	93	8 (2)	In
Overall quality of life	0	8	92	0	14	86	89	8 (2)	In
Overall health status	0	16	84	3	21	76	80	8 (2)	In
Fatigue	0	28	72	0	24	76	74	7 (3)	In
Cognitive problems	0	20	80	0	31	69	74	7 (2)	In
Functional impairment	4	28	68	3	17	79	74	7 (2)	In
Financial toxicity	0	16	84	0	41	59	70	7 (3)	In
Fear of cancer recurrence or progression	4	28	68	3	28	69	69	7 (2)	Marginal
Role limitations	0	32	68	0	34	66	67	7 (2)	Marginal
Impact on family and relationships	4	28	68	10	31	59	63	7 (1)	Marginal
Psychological adjustment to cancer	4	24	72	3	48	48	59	7 (2)	Marginal
Peripheral neuropathy	4	24	68	3	48	48	57	7 (1)	Marginal
Mobility	4	28	64	0	41	59	61	7 (2)	Out
Sleep disturbance	4	36	60	0	48	52	56	7 (2)	Out
Social support	0	48	52	3	41	55	54	7 (3)	Out
Access to supportive care and services	0	40	60	10	41	48	54	7 (3)	Out
Health service utilisation	4	32	64	10	45	45	54	7 (1)	Out
Work-related distress	4	32	64	7	55	38	50	6.5 (2)	Out
Social limitations	0	52	48	3	48	48	48	6 (3)	Out
Bowel problems	4	44	48	3	48	48	48	6.5 (2)	Out
Lymphedema	4	28	60	7	55	38	48	6.5 (2)	Out
Incontinence	4	36	60	3	59	38	48	6 (1)	Out
Fertility and reproductive problems	4	40	52	0	59	41	46	6 (1)	Out
Existential distress	12	28	60	17	48	34	46	6 (2)	Out
Cultural and linguistic barriers to obtaining, accepting, and understanding treatments	4	32	60	10	35	34	46	6 (1)	Out
Sexual functioning	8	52	40	0	52	48	44	6 (2)	Out
Social isolation	4	52	44	7	52	41	43	6 (2)	Out
Lifestyle modifications	12	40	48	10	52	38	43	6 (1)	Out
Health literacy	8	40	52	3	62	34	43	6 (2)	Out
Dyspnoea (i.e., breathlessness)	8	52	36	3	52	45	41	6 (2)	Out
Satisfaction with care received	12	28	60	10	6	24	41	6 (3)	Out
Informational support	12	44	44	3	62	34	39	6 (1)	Out
Nausea/vomiting	4	44	48	3	66	31	39	6 (3)	Out
Cachexia (i.e., muscle weakness)	8	52	36	0	62	38	37	6 (2)	Out
Changes in body weight	8	52	40	0	66	34	37	6 (2)	Out
Practical difficulties	8	40	48	28	45	28	37	6 (3)	Out
Empowerment	4	40	56	21	59	21	37	6 (2)	Out
Body image	8	52	40	10	59	31	35	6 (3)	Out
Sense of identity	12	48	40	17	52	31	35	6 (2)	Out
Pressures after cancer	8	44	48	7	69	24	35	6 (2)	Out
Appetite loss	12	56	32	7	59	34	33	6 (2)	Out
Dysphagia (i.e., swallowing difficulty)	12	44	40	0	72	28	33	6 (2)	Out
Sense of dignity	8	60	32	10	66	24	28	6 (2)	Out
Cancer-related stigma and marginalisation	12	48	40	24	59	17	28	6 (3)	Out
Altered vision	12	48	36	10	76	14	24	6 (3)	Out
Positive changes	16	48	36	24	62	14	24	6 (3)	Out
Altered hearing	12	56	28	10	72	17	22	6 (3)	Out
Altered speech	16	44	36	17	76	7	20	6 (2)	Out
Spirituality	24	60	16	34	45	21	19	5 (3)	Out
Altered taste	16	60	20	21	69	10	15	5 (2)	Out

Outcomes were classified as ‘In’ if they met the following consensus criteria: (i) median score ≥ 7 with an interquartile range no greater than 2 units, (ii) rated between 7 and 9 by at least 70% of panellists, (iii) rated between 1 and 3 by less than 15% of panellists. Outcomes were ‘Marginal’ if they had a median score ≥ 7 and ≥ 65% majority agreement for one panel. Outcomes that did not meet these criteria were classified as ‘Out’

^1^Allocation of scores of 1–9 displayed only; ratings of ‘Unable to score’ not shown

#### Consensus meeting

The consensus group meeting was facilitated by IR and attended by 16 Delphi participants, including 4 members of the research team. Four participants were researchers, including a behavioural scientist, a PROs specialist, a cancer epidemiologist, and a policy researcher. Four participants were cancer survivors, including one researcher. Nine participants were clinicians or clinician researchers who practiced in the fields of nursing, medical oncology, radiation oncology, clinical psychology, and speech pathology. Of these, 3 also had a health policy background. There was unanimous agreement on the inclusion of the following outcome domains in the core outcome set without any suggested changes: depression, anxiety, fear of cancer recurrence or progression, cognitive problems, financial toxicity, pain, fatigue, overall health status, and overall quality of life. Although there was also agreement on the inclusion of functional impairment and role limitations, overlap between these concepts was flagged as a conceptual issue. All participants agreed on the exclusion of peripheral neuropathy on the basis that it was too specific and that its consequences could be captured by other domains (e.g., pain, functional impairment, and/or symptom burden).

The presence and trajectory of symptoms and late effects during survivorship were considered important to capture at a population level. However, the length of symptom burden measures, their differing conceptualisations of symptom burden (i.e., the presence, severity, frequency, or interference of symptoms), and interpretability of this domain were issues. Similarly, concerns were raised regarding how the domains of psychological adjustment to cancer and impact on family and relationships would be operationalised, measured, and interpreted by consumers. It was decided that further conceptual clarification in response to the concerns raised was required, to ensure that the domains could be operationalised before finalising the core outcome set.

Based on the concerns and suggestions raised in the meeting, the terminology and operational definitions of four domains were refined by examining how outcomes had been defined, operationalised, and measured in the PROMs literature. Functional impairment and role limitations were combined into a single domain focused on ability to perform and participate in daily activities including functioning in usual social and occupational roles. Overall symptom burden was reframed to ‘overall bother from side effects’ to distinguish it from comprehensive measures of symptom burden and address concerns about its interpretability to consumers. This was considered conceptually clear and amenable to a single item. In response to similar concerns about interpretability, psychological adjustment to cancer was reframed as ‘coping with cancer’ to capture the behavioural and cognitive responses that cancer survivors use in their efforts to adjust to cancer and avoid conflation with the presence of adjustment disorders. The availability of suitable measures for assessment of this domain remains a potential challenge. A clear conceptual and operational definition for the domain assessing the impact of cancer on family, relationships, and parenting could not be determined based on the literature.

The provisional core outcome set presented in Table 3 was approved by 14 out of 16 meeting attendees. One dissenting participant objected to the exclusion of the domain focused on the impact of cancer on family and relationships. The other dissenting participant acknowledged that all the proposed domains were important but anticipated that the burden of collecting them would be too great for the core outcome set to be used routinely. No objections were raised regarding specific included domains. It was thus deemed appropriate to progress the provisional core outcome set (outlined in Table 3) to phase III, where suitable measures for its assessment will be identified. The issues raised by dissenting participants are discussed in more detail in the discussion and will be considered in phase III.

**Table 3:** Agreed domains of the cancer survivorship core outcome set and their lay definitions

1. Depression—persistent feelings of sadness, helplessness, or loss of enjoyment	
2. Anxiety—worry, dread, fear, or unease	
3. Pain—an unpleasant sensory and emotional experience associated with actual or potential tissue damage	
4. Fatigue—feeling weak, lacking in energy, tired, drained, or exhausted	
5. Cognitive problems—loss of cognitive abilities such as memory, thinking, attention, or multi-tasking	
6. Fear of cancer recurrence or progression—fear that cancer could return or progress in the same place or another part of the body	
7. Functioning in usual activities and roles—the ability to perform and participate in daily activities, including functioning in usual social and occupational roles	
8. Financial toxicity—financial hardship or distress associated with concerns about the costs of cancer and its impact on income	
9. Coping with cancer—the behavioural and cognitive responses that people use in their efforts to adjust to cancer	
10. Overall bother from side effects—the extent to which someone is bothered by side effects from cancer and the associated disruption to normal activities	
11. Overall quality of life—a general indicator of life satisfaction and physical, emotional, and social well-being	
12. Overall health status—a general indicator of health and the presence of disease	

## Discussion

This study used robust methods to generate a core outcome set for national surveillance of the long-term quality of life of cancer survivors in Australia. After two survey rounds and a group consensus meeting with national experts in cancer survivorship practice, research, policy, advocacy, and lived experience, 12 core domains were agreed upon. These were depression, anxiety, pain, fatigue, cognitive problems, fear of cancer recurrence or progression, functioning in everyday activities and roles, financial toxicity, coping with cancer, overall bother from side effects, overall quality of life, and overall health status.

The prioritisation of outcomes in the consensus meeting was driven by several key considerations that emerged as themes in the discussion. Firstly, it was considered important to capture outcomes not well represented in other cancer datasets and with a relatively unknown prevalence at the population level. Secondly, feasibility for the intended application of population surveillance was central to the discussion and prompted considerations regarding the availability, potential overlap, and combined length of prospective measures. Ensuring clear operational definitions for each domain and including broad summary items (e.g., overall quality of life), which could add useful information with little added respondent burden, were agreed avenues for optimising feasibility. Finally, the inclusion of positive and negative items in the core outcome set was deemed important to inform understanding of not only the impacts of cancer, but individual factors that may influence outcomes.

Our core set and the core set developed by the NCI [15] share 5 outcome domains in common: pain, fatigue, anxiety, depression, and cognitive problems. Similar outcomes are also represented in the core set developed by Geerse and colleagues [16], including sensation of pain, energy and drive functions, attention functions, and emotional functions. This commonality reflects the broader literature which indicates that pain, fatigue, cognitive limitations, anxiety, and depressive symptoms are consistently present in cancer survivors following primary treatment [13]. In addition to these, the present core outcome set includes fear of cancer recurrence or progression, functioning in usual activities and roles, financial toxicity, coping with cancer, and overall indicators of health status, quality of life, and bother from side effects. The prioritisation of these domains is consistent with literature highlighting the prevalence of issues related to daily functioning [29], fear of cancer recurrence [30], and financial toxicity (due to mounting medical expenses, lost wages, and reduced productivity) among cancer survivors [31]. The NCI set captures more treatment-related symptoms (e.g., diarrhoea, constipation, dyspnoea, sensory neuropathy) [15], while the core set developed by Geerse and colleagues includes several environmental factors (e.g., friends; immediate family; health professionals; social security series, systems, and policies) and more specific functional domains (e.g., reading, driving, sexual functions) [16].

Defining and operationalising multidimensional and subjective constructs were challenging aspects of this exercise, particularly when supporting evidence from the PROMs literature was limited or discordant. For this reason, the panel could not determine consensus on a domain representing the impact of cancer on relationships and families, although this was evidently an important outcome. The issue with this domain was how broadly it could be interpreted and how multifaceted and multidirectional the impacts of cancer on families and relationships could be. This is apparent from the limited survivorship PROMs that assess wide-ranging aspects of this domain, such as marital communication, affection, neglect, and overprotection; parental concerns about both the practical and emotional impacts of cancer on the child; parental efficacy beliefs; support from and communication with close relatives; perceived impacts of cancer on caring for family members, attending family events, time spent with family, and plans to have a family; and interference of illness with family relationships and couple intimacy [32]. The impact of cancer on family roles, responsibilities, and relationships, and the measurement of these constructs, are areas requiring further research.

The challenge of quantifying the impacts of cancer on relationships was also highlighted in the core outcome set developed by Geerse and colleagues [16], which included the two related domains ‘basic interpersonal interactions’ and ‘complex interpersonal interactions’. During the process of matching their core outcome domains to questionnaire items from existing PROMs, the authors linked the ‘basic interpersonal interactions’ domain to three items on the Quality of Life in Adult Cancer Survivors (QLACS) [33] questionnaire and one item on the Impact of Cancer Version 2 (IOCv2) [34] questionnaire; and the ‘complex interpersonal interactions’ domain to two items on QLACs, 8 items on IOCv2, and three items on the Distress Thermometer and Problems List (DT/PL) [35]. Although the questionnaire items are not specified, it is evident from examining the measures that the aspects of interactions they assess differ. QLACS contains a ‘social avoidance’ subscale that assesses reluctance to start new relationships and meet new people, and avoidance of social gatherings and friends, as well as a ‘family distress’ domain that assesses worry about family members being at risk for cancer, having cancer-causing genes, or having to undergo genetic testing for cancer [33]. IOCv2 contains a ‘life interferences’ subscale that captures guilt for not being available to family, feeling alone, feeling misunderstood, and the interference of cancer with usual activities, as well as a ‘relationship concerns’ subscale that assesses self and partner openness and willingness to discuss cancer, relationship problems due to health uncertainty, and worry about being left by partner if cancer was to return [34]. DT/PL contains a ‘family problems’ scale that assesses problems related to dealing with children, dealing with partner, ability to have children, and family health issues [35]. Application of the core outcome set may therefore result in conceptually different constructs being assessed depending on the measure(s) used. This example illustrates why further exploration and conceptual clarification of the interpersonal impacts of cancer and their measurement are required.

We based the study methods on recommendations for evidence-based core outcome set development [25], guidelines for using the Delphi technique to obtain consensus on core outcomes [36], standards for core outcome set study design [22], and previous core outcome set studies with cancer populations that employed Delphi methods [27, 37]. However, there is no agreed-upon methodology for developing a core outcome set. It is therefore unclear to what extent the results from this study would be concordant with those obtained in different settings, using alternate consensus methods, or applying different criteria. Despite these limitations, the study design was considered suitable for the scope and setting of this core outcome set and allowed a large and geographically diverse sample of stakeholders to participate.

Although there is no consensus on the optimal number of Delphi rounds, two or three rounds have been frequently recommended [38] and commonly used in core outcome set development studies [25]. Given that we undertook a rigorous process to identify and refine the initial list of outcomes for the Delphi study and included a consensus meeting, two questionnaire rounds were considered sufficient. An advantage of restricting the number of rounds is limiting potential bias due to attrition, which is likely to increase with each round [36]. A limitation of restricting the study to two questionnaire rounds is that it was not possible to confirm stability of voting, although this is generally thought to be a measure of internal reliability and not consensus [39]. Instead, we measured the extent to which participants agreed with the statements under consideration (agreement) and the extent to which participants agreed with each other (consensus) [40]. It is not possible to determine the validity of any specific definition of consensus in Delphi studies, but the proportion of ratings within a range is one of the most commonly employed consensus definitions and the median is considered the most robust measure of central tendency [40].

Two minor deviations from our study protocol were made for pragmatic reasons. Firstly, because the focus group discussions generated an extremely comprehensive list of outcomes, we elected to conduct a rapid and targeted search for existing review evidence rather than an exhaustive literature review of cancer survivorship PROMs. While it is therefore possible that not all relevant PROMs were identified, the extensive overlap between the outcomes derived from the focus groups, PROMs, and guiding PRO framework suggests that this would not have resulted in any relevant outcome domains being missed. This risk was further mitigated by providing Delphi participants with the opportunity to suggest additional outcomes. Secondly, although we planned to have three stakeholder panels participate in the Delphi survey (cancer survivors, clinicians, and other healthcare and research professionals) and invited a proportionate number of experts accordingly, response was significantly higher among cancer survivors than the other invited groups. Since cancer survivors comprised half of our recruited sample in round 1, we made the decision to group participants into two equally sized panels rather than three. This made it easier to compare results by group and did not compromise the study findings beyond giving additional weight to the views of consumers.

Further work is required to determine how the core outcome set will be assessed and applied in practice. In phase III, valid and reliable measures for assessment of the core outcome domains will be identified, appraised, and selected with expert advice and input from end users [18]. The final core measurement set will be piloted to ensure its acceptability to cancer survivors and feasibility for routine collection at a population level. As noted during the consensus meeting, further refinement of the core outcome set may occur during this phase of the research and over time as we learn more about the overlap among measures and usefulness of individual concepts.

## Conclusion

This study describes the development of a core set of patient-reported outcomes, representing the most prevalent and important issues that experts agree should be assessed as a minimum in population-based research on cancer survivorship. Since the core outcome set represents only the minimum outcomes that should be collected and reported on, it can be supplemented with other outcomes or measures relevant to a given study setting or population. By delivering consensus-driven recommendations that reflect consumer, health professional, research, and policy perspectives, the study findings will facilitate the inclusion of meaningful survivorship outcomes in future studies. Adoption of the core outcome set will enhance the quality and comparability of PRO data collected in survivorship research, particularly when applied to address macro-level questions.

## Abbreviations

COS-STAD: Core Outcome Set-Standards for Design
COS-STAR: Core Outcome Set-Standards for Reporting
DT/PL: Distress Thermometer and Problems List
GRADE: Grading of Recommendations Assessment, Development and Evaluation
ICF: International Classification of Functioning, Disability and Health
IOCv2: Impact of Cancer Version 2
NCI: National Cancer Institute
PRO(s): patient-reported outcome(s)
PROM(s): patient-reported outcome measure(s)
QLACS: Quality of Life in Adult Cancer Survivors
